# IgG-index predicts neurological morbidity in patients with infectious central nervous system diseases

**DOI:** 10.1186/1471-2334-10-202

**Published:** 2010-07-09

**Authors:** Peter Lackner, Elif Guengoer, Ronny Beer, Gregor Broessner, Raimund Helbok, Florian Deisenhammer, Erich Schmutzhard, Bettina Pfausler

**Affiliations:** 1 Department of Neurology, Innsbruck Medical University, Austria

## Abstract

**Background:**

Prognosis assessment of patients with infectious and neoplastic disorders of the central nervous system (CNS) may still pose a challenge. In this retrospective cross-sectional study the prognostic value of basic cerebrospinal fluid (CSF) parameters in patients with bacterial meningitis, viral meningoencephalitis and leptomeningeal metastases were evaluated.

**Methods:**

White blood cell count, CSF/serum glucose ratio, protein, CSF/serum albumin quotient and Immunoglobulin indices for IgG, IgA and IgM were analyzed in 90 patients with bacterial meningitis, 117 patients with viral meningoencephalitis and 36 patients with leptomeningeal metastases in a total of 480 CSF samples.

**Results:**

In the initial spinal tap, the IgG-index was the only independent predictor for unfavorable outcome (GOS < 5) in patients with infectious CNS diseases but not in patients with leptomeningeal metastases. The sensitivity and specificity of an IgG-index of 0.75 and higher for predicting unfavorable outcome was 40.9% and 80.8% in bacterial meningitis and 40% and 94.8% in viral meningoencephalitis, respectively. No significant associations between CSF parameters and outcome could be observed in follow-up CSF samples.

**Conclusion:**

The present study suggests that in infectious CNS diseases an elevated IgG-Index might be an additional marker for the early identification of patients at risk for neurological morbidity.

## Background

The clinical course of infectious and neoplastic disorders of the central nervous system is sometimes difficult to predict. While the diagnosis of bacterial meningitis, viral meningitis/meningoencephalitis and leptomeningeal metastases (LM) mainly relies on the analysis of cerebrospinal fluid (CSF), only limited data on the prognostic value of CSF parameters exist [1]. Yet, initial risk assessment of individual patients is of paramount importance in order to choose the appropriate level of further surveillance (i.e. general ward versus critical care unit) [2]. Of course, clinical presentation is one of the most important issues in this respect [3]. This has been shown by different authors and complex scores have been developed in order to raise the predictive accuracy of clinical signs and symptoms [2,4-7]. In addition, other studies have tried to assess the role of imaging techniques such as computed tomography or transcranial Doppler sonography [8,9]. Despite these improvements in clinical and imaging workup, significance of basic CSF analyses for the early identification of patients at risk for neurological morbidity has not been sufficiently evaluated. Moreover existing studies were performed only in one or the other of the above mentioned disease entities. However, in the early course of the disease the differential diagnosis of inflammatory CNS diseases is not always easy. Therefore this retrospective study was conducted in order to evaluate the predictive power of basic CSF parameters obtained by the initial as well as follow-up spinal taps for disease prognosis in patient with bacterial meningitis, viral meningitis/meningoencephalitis and LM.

## Methods

### Patients

Over a period of 12 years (January 1996 through September 2007) all patients requiring lumbar puncture for differential diagnosis of neurological diseases were queried from the central CSF database of the Department of Neurology. A total of 1675 patients were found. Only patients with CSF pleocytosis (more than 4 leukocytes/mm^3^) showing less than 7000 red blood cells/mm^3 ^were eligible for further analysis (n = 835). Of these 835 patients, 592 patients had to be excluded due to insufficient data or inconclusive diagnosis. Finally, 243 patients remained in the data set. In these patients a total of 480 CSF samples were collected. Patients were stratified into 3 diagnostic groups (bacterial meningitis, viral meningoencephalitis, LM) diagnosed by commonly accepted clinical and/or microbiological and pathological/cytological criteria [10].

### Data collection and outcome measure

The following CSF variables were included in the analyses: white blood cell count (WBC), CSF/serum glucose ratio (GluR), CSF/serum albumin quotient (Qalb), indices for IgG, IgA and IgM (IgG-, IgA-, IgM-index representing the CSF/serum Ig ratio in relation to Qalb) [1]. Intrathecal immunoglobulin synthesis was calculated as described by Reiber et al. [11]. The neurological outcome at discharge (Glasgow outcome scale, GOS) was evaluated by chart review. The GOS grades neurological outcome on a scale from 1 to 5. A score of 1 indicates death; 2, persistent vegetative state (the patient is unable to interact with the environment); 3, severe disability (the patient is unable to live independently but can follow commands); 4, moderate disability (the patient is capable of living independently but unable to return to work or school); and 5, mild or no disability (the patient is able to return to work or school).

GOS was dichotomized to receive binary outcome measures for logistic regression analyses into unfavorable outcome (GOS 1-4) and favorable outcome (GOS 5) [12]. All data acquisition was done by purely retrospective chart review. Subsequent analyses were performed on anonymized data and therefore institutional review board approval was waived due to Austrian regulations.

### Statistical methods

Patient characteristics were compared between diagnostic groups by ANOVA (age, length of stay (LOS)) or chi-square test (GOS, sex, confirmed etiology) depending on the data type and distribution. For outcome analysis a forward conditional stepwise binary logistic regression model was calculated using dichotomized GOS as dependent and the respective CSF parameters of the first spinal tap of each patient as independent variables. Raw data of all parameters were entered into the model on a non selective basis. In the stepwise procedure, a significance level of 0.05 for entering and 0.10 for removing the respective explanatory variables was set. Age and primary diagnoses were included as mandatory predictors. In addition univariate binary logistic regression models were calculated of CSF parameters in each patient group. Finally, multivariate logistic regression models with the significant predictors of the univariate and the stepwise procedure as well as age and Qalb as important covariates were calculated. The levels of the IgG-index in follow-up CSF samples were analyzed by Wilcoxon signed rank sum test and p-values were Bonferroni corrected for multiple comparisons. Sensitivity and specificity of the IgG-index for unfavorable outcome was determined by drawing ROC curves and the cut-off value of 0.75 was chosen to reach maximum specificity. Correlations were calculated by Spearman's rank correlation. Calculations were done using PASW 18 (IBM, Chicago, IL, USA). Graphs were drawn in part with GraphPad Prism 5.00 (GraphPad Software, San Diego, CA, USA).

## Results

### Patients' characteristics

Among 243 patients with inflammatory CSF, 90 patients were diagnosed with bacterial meningitis, 117 patients with viral meningoencephalitis and 36 patients with LM, respectively (table 1).

**Table 1:** Patient characteristics

Patient characteristics				
**characteristic**	**bacterial meningitis**	**viral meningoencephalitis**	**leptomeningeal metastases**	**p**

no. of patients	90	117	36	
mean age in years (SD)	48.1 (19.5)	39.8 (17)	52.8 (12)	p < 0.001
length of stay in days (SD)	19.3 (23)	10.6 (19.4)	17.3 (17.9)	p = ns
female patients (%)	41.1	40.3	50	p = ns
				
GOS (%)				p < 0.001
5 (fully independent)	67.8	94	13.9	
4 (moderate disability)	15.6	5.1	25	
3 (severe disability)	6.7	0	19.4	
2 (persistent vegetative state)	2.2	0.9	5.6	
1 (dead)	7.8	0	36.1	
				
confirmed etiology (%)	62.2	17.1	100	p < 0.001

The etiology was confirmed by microbiology, serology and/or PCR in 62.2% of the patients with bacterial meningitis and 17.1% of the patients with viral meningoencephalitis (table 1). The most frequent causative microorganism in bacterial meningitis was Streptococcus pneumoniae and tick borne encephalitis virus in patients with viral meningoencephalitis (table 2). LM was diagnosed by CSF cytology.

**Table 2:** Etiology

Etiology		
		
	
	**no. of cases**	**%**

**BACTERIAL**		
Streptococcus pneumoniae	25	44.6
Neisseria meningitidis	9	16.1
Mycobacterium tuberculosis	8	14.3
Borrelia burgdorferi	3	5.4
Hemophilus influenzae	3	5.4
Staphylococcus aureus	3	5.4
Streptococcus spp.	3	5.4
Listeria moncytogenes	1	1.8
Multiple microorganisms	1	1.8
		
**VIRAL**		
Tick borne encephalitis virus	12	60
Varicella Zoster virus	4	20
Cytomegalic virus	1	5
Epstein-Barr virus	1	5
Herpes simplex virus	1	5
Mumps virus	1	5
		
**LM (primary tumor)**		
Breast cancer	13	36.1
Lung cancer	11	30.6
B-cell lymphoma	3	8.3
Primitive neuroectodermal tumor	2	5.6
B-CLL	1	2.8
Bladder cancer	1	2.8
Glioblastoma multiforme	1	2.8
Multiple myeloma	1	2.8
Renal cell carcinoma	1	2.8
Squamous cell carcinoma	1	2.8
AML	1	2.8

A total of 480 CSF samples were collected. In 95 patients (39.1%) at least two CSF samples were analyzed. In 39 patients (16.0%) a third CSF sample was available. The median time gap and interquartile range (IQR) between the first and second or the second and the third spinal tap respectively was 5/7 days (IQR 8/6) in patients with bacterial meningitis, 7/13.5 days (IQR 3/7) in patients with viral meningoencephalitis and 2/3 days (IQR 3/5) in patients with LM. These differences were statistically significant (p < 0.01). The mean age was significantly different between groups, being lowest in patients with viral meningoencephalitis and highest in patients with LM (p < 0.001, table 1). Length of stay and percentage of female patients did not differ significantly between groups (table 1). Outcome, measured by GOS, showed statistically significant differences between the patient groups. 94% of the patients with viral meningoencephalitis and 62.2% of the patients with bacterial meningitis but only 13.9% of LM patients showed a full recovery or only mild neurological deficits at first discharge (p < 0.001, table 1).

### Outcome analyses

**Figure 1:**
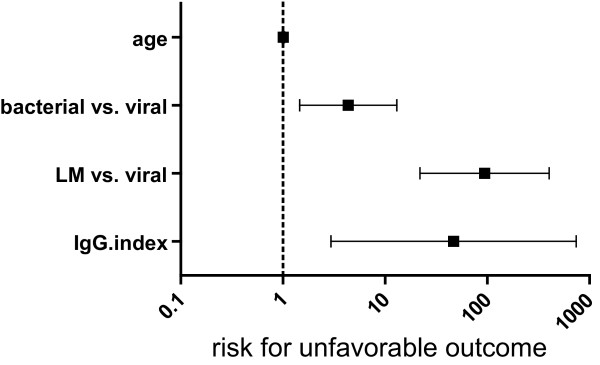
**Outcome analyses**. Risk for unfavorable outcome (GOS < 5). Odds ratios with confidence interval from a logistic regression model are shown (leptomeningeal metastases, LM). Patients with bacterial meningitis and LM showed an increased risk for unfavorable outcome compared to patients with viral meningoencephalitis. The IgG-index was an independent predictor for unfavorable outcome (OR 46.75, CI 2.95-740.14).

**Figure 2:**
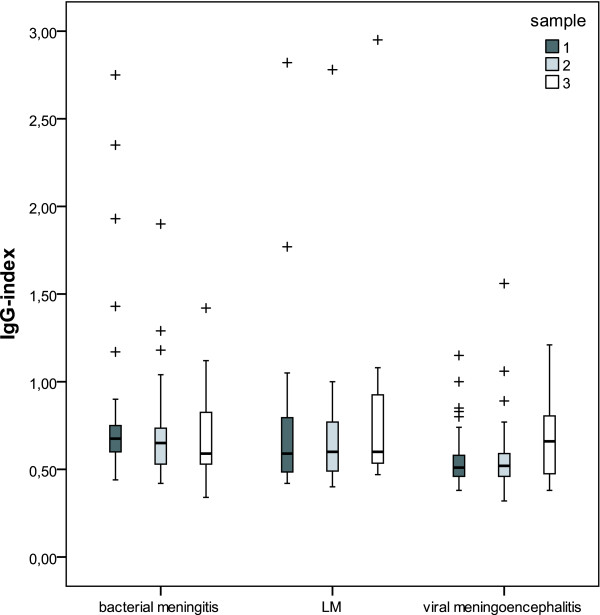
**Time course of the IgG-index in follow-up CSF samples**. The time course of the IgG-index in the initial and follow-up CSF samples is shown in patients with bacterial meningitis, leptomeningeal metastases and viral meningoencephalitis. The median time gap and interquartile range (IQR) between the first and second or the second and the third spinal tap respectively was 5/7 days (IQR 8/6) in patients with bacterial meningitis, 7/13.5 days (IQR 3/7) in patients with viral meningoencephalitis and 2/3 days (IQR 3/5) in patients with LM.

A stepwise logistic regression model with the dichotomized GOS as dependent and age, primary diagnoses as well as the respective CSF parameters as independent variables was calculated. The only CSF parameter showing a significant odds ratio (OR) in this procedure was the IgG-index. The final model is shown in figure 1. Patients with bacterial meningitis and LM had an increased risk for unfavorable outcome as compared to patients with viral meningoencephalitis. The IgG-index was an independent predictor of unfavorable outcome (OR 46.75, CI 2.95-740.14). When including only patients with confirmed etiology (n = 112), primary diagnoses and IgG-index still showed a significant association with outcome (IgG-Index: OR 25.86, CI 1.31 - 510.64). To further verify the predictive value of the IgG-index, logistic regression models for each patient group were calculated (table 3). In the univariate approach a significant OR for the prediction of unfavorable outcome was found for all immunoglobulin indices in viral meningoencephalitis and for the IgG-index in bacterial meningitis (table 3). In the multivariate approach besides age, Qalb was included to correct for blood brain barrier (BBB) dysfunction. In these models only the IgG-index remained an independent predictor of outcome in patients with bacterial meningitis and viral meningoencephalitis but not in patients with LM. Statistically significant correlations between the IgG-index and CSF parameters indicating BBB dysfunction (Qalb and protein) were observed in patients with bacterial meningitis (Qalb: rho = 0.52, p < 0.001; protein: rho = 0.54, p < 0.001) and patients with LM (Qalb: rho = 0.39, p = 0.04; protein: rho = 0.60, p = 0.01), but not in patients with viral meningoencephalitis (Qalb: rho=-0.13, p = 0.21; protein: rho=-0.05, p = 0.62). The sensitivity and specificity of an IgG-index of 0.75 and higher to predict unfavorable outcome was 40.9% and 80.8% in bacterial meningitis and 40% and 94.8% in viral meningoencephalitis, respectively. Intrathecal immunoglobulin synthesis in patients with an IgG-index of 0.75 and higher was observed in 26.3% of patients with bacterial meningitis, 55.6% of patients with LM and 100% of patients with viral meningoencephalitis. Absolute values for intrathecal immunoglobulin response did not show an association to outcome. When analyzing only the second spinal tap or the change of the respective parameters between the first and second spinal tap no significant association with outcome could be found. In patients with bacterial meningitis the levels of the IgG-index showed a gradual, statistically non-significant, decrease in follow up CSF samples (figure 2). There were no changes of the mean levels of the IgG-index in LM patients. In patients with viral meningitis the IgG-index increased in the third CSF sample however also this difference was not statistically significant.

**Table 3:** Outcome analyses

Prediction of unfavorable outcome (GOS < 5)						
	**bacterial meningitis**		**viral meningo-encephalitis**		**leptomeningeal metastases**	

**Univariate Models**						
	**OR (CI)**	**p-value**	**OR (CI)**	**p-value**	**OR (CI)**	**p-value**
			
leukocyte count	0.999 (0.999-1.000)	0.061	0.998 (0.992-1.003)	0.551	0.998 (0.996-1.000)	0.147
GluR	0.664 (0.193-2.282)	0.516	0.166 (0.003-8.677)	0.374	0.023 (0.000-21.15)	0.280
protein	0.999 (0.998-1.000)	0.560	1.006 (0.989-1.023)	0.460	1.000 (0.998-1.002)	0.841
Qalb	1.002 (0.991-1.013)	0.674	1.067 (0.988-1.152)	0.095	1.002 (0.986-1.019)	0.761
IgM-index	0.927 (0.178-4.818)	0.929	14.71 (1.020-212.0)	**0.048**	0.800 (0.028-22.20)	0.896
IgA-index	1.902 (0.190-19.02)	0.584	655.4 (2.56-167554)	**0.022**	0.751 (0.015-37.38)	0.886
IgG-index	111.1 (1.876-6587)	**0.024**	539.8 (3.605-80821)	**0.014**	1.371 (0.097-19.20)	0.815
**Multivariate Model**						
	**OR (CI)**	**p-value**	**OR (CI)**	**p-value**	**OR (CI)**	**p-value**
			
age	1.004 (0.974-1.034)	0.801	1.015 (0.965-1.069)	0.565	0.970 (0.882-1.067)	0.529
Qalb	0.995 (0.981-1.009)	0.547	1.084 (0.993-1.184)	0.071	1.000 (0.984-1.017)	0.680
IgG-index	182.1 (1.6-20143)	**0.030**	767.6 (3.5-167197)	**0.016**	1.3 (0.114-15.806)	0.816

## Discussion

The current study was conducted to evaluate the prognostic value of basic CSF parameters in patients with bacterial meningitis, viral meningoencephalitis and LM. The only CSF parameter of prognostic significance was the IgG-index. An IgG-index of 0.75 and higher was found to be a highly specific predictor of unfavorable outcome in patients with bacterial and viral meningoencephalitis but not in patients with LM.

The importance of basic CSF parameters has been studied in detail and guidelines for the use of routine CSF analyses in the differential diagnosis of CNS diseases are available [1]. In this respect the current study gave similar results. Besides differential diagnosis, risk assessment in the early phase of diagnostic work-up and therapeutic management is of paramount importance. In particular clinical factors like focal neurological deficits and impaired consciousness but also paraclinical data are of value [4,6]. However, recent investigations have focused on risk assessment in patients with only one predefined diagnosis. In the current multivariate approach, all patients with inflammatory CSF were analyzed together including important covariates. Interestingly, besides primary diagnosis, the IgG-index was the only independent predictor of unfavorable outcome. In bacterial meningitis another study showed similar results [13]. Although only a low number of patients were included in the mentioned study, patients with neurological morbidity due to bacterial meningitis showed a significantly higher IgG-index in the initial CSF than patients who fully recovered [13]. In addition to pathogen virulence, immune mediated mechanisms are discussed as important determinants of outcome in inflammatory CNS disease. Therefore, in bacterial meningitis early adjunctive administration of corticosteroids has been suggested. Steroids might support adrenal insufficiency as well as reduce antimicrobial-induced inflammatory response [12,14], however a recent meta-analysis could not show a positive effect on mortality or morbidity [15]. Hence this adjunctive measure and in particular its putative effect on the reduction of host immune response is still not clear and requires further studies.

While steroid treatment primarily aims at inhibiting innate immune mechanisms which are certainly most important in the early phase of bacterial meningitis [16,17], antibody mediated host response may be detrimental as well. This is supported by a recent paper showing early accumulation of dendritic cells in the CSF during bacterial meningitis which might lead to rapid induction of adaptive immune mechanisms [18]. Therefore the observed association of an elevated IgG-index with unfavorable outcome may be explained by secondary neuronal damage due to early intrathecal immunoglobulin reaction. In the current study, IgG-index levels gradually decreased in patients with bacterial meningitis during the hospital stay. Furthermore, no association of CSF parameters with outcome could be observed in follow up CSF samples. These findings support the hypothesis of brain damage by overwhelming host immune response in the early phase of meningitis. Adaptive immune response to different pathogens is highly specific [19] and can certainly not be compared between infectious and neoplastic CNS diseases. In the current study an elevated IgG-index was an independent predictor of unfavorable outcome only in patients with infectious CNS disease but not in the LM group. This could indicate that specific microorganism-driven immunoglobulin production is more likely to cause inflammatory brain damage resulting in neurological morbidity than response to neoplastic cells in LM [20]. However, the association of an elevated IgG-index with unfavorable outcome could also be explained as an unspecific epiphenomenon of brain damage. Yet the exact pathomechanisms remain elusive and require further studies.

The current study shows that an elevated IgG-index predicts unfavorable outcome in patients with bacterial and viral meningoencephalitis but not in patients with LM. In patients with bacterial meningitis blood brain barrier damage is immanent. An elevated IgG-index in these patients could at least in part be attributable to BBB disruption. This is supported by the observed correlation between the IgG-index and Qalb and total CSF protein. However such correlations were not observed in patients with viral meningoencephalitis in whom the OR and the specificity of an elevated IgG-index for the prediction of unfavorable outcome were even higher than in bacterial meningitis. This suggests that an elevated value for IgG in CSF is associated with bad outcome irrespective of BBB integrity.

It should be noted that the average time to result for immunoglobulin indices with modern analyzers is well below half an hour. Therefore this rapid test is easily applicable in the emergency setting and the current data suggests that patients with an IgG-index of 0.75 and above at initial presentation should receive special attention.

Like in every retrospective investigation, also the current data has shortcomings which have to be kept in mind when interpreting the results. Primary etiology was not confirmed in some patients with bacterial meningitis. In patients with viral meningoencephalitis the percentage with confirmed etiology was low compared to reports by others [21]. This is most likely attributed to the fact that patients with viral meningitis, who are quickly recovering, are not routinely followed-up serologically at the study hospital due to the lack of therapeutic consequences and economic reasons. In these patients clinical presentation, laboratory values and also CSF analyses influenced the final diagnosis. Due to this potential a-priori selection bias we have computed regression models including only patients with confirmed etiology. The results were comparable, though the statistical power was lower due to the lower number of cases. Therefore we are convinced that the results of this study are reliable.

## Conclusion

In conclusion, an elevated IgG-Index might be added to the list of markers for the early identification of patients at high risk for neurological morbidity in infectious CNS diseases. Further prospective studies are warranted to verify the present findings and to evaluate the prognostic potential of the IgG-index in combination with other clinical and laboratory data.

## Competing interests

The authors declare that they have no competing interests.

## Authors' contributions

PL, ES and BP had the idea and designed the study. EG, GB and RH performed all data acquisitions. PL, RB, BP and FD analyzed the data and drafted the manuscript. All authors participated in the interpretation, and writing up of the research work and read and approved the final manuscript.

## Funding

This work was supported by internal funding of Innsbruck Medical University.

## Pre-publication history

The pre-publication history for this paper can be accessed here:

http://www.biomedcentral.com/1471-2334/10/202/prepub
